# Assessment of Iodine Status in Children, Adults, Pregnant Women and Lactating Women in Iodine-Replete Areas of China

**DOI:** 10.1371/journal.pone.0081294

**Published:** 2013-11-25

**Authors:** Fangang Meng, Rencheng Zhao, Peng Liu, Lixiang Liu, Shoujun Liu

**Affiliations:** Institute of Iodine Deficiency Disorders, Center for Endemic Disease Control, Chinese Center for Disease Control and Prevention, Harbin Medical University, Harbin, People’s Republic of China; National Institute for Viral Disease Control and Prevention, China

## Abstract

**Background:**

Iodine deficiency disorders (IDD) are widespread in China. Presently, IDD have been put under control by Universal Salt Iodisation (USI) in China; however, there is a lack of evidence on whether the iodine status in adults, pregnant women and lactating women is optimal. This study was therefore conducted to assess the iodine nutrition and thyroid function of children, adults, pregnant women and lactating women residing in areas where the USI program is fully established.

**Design:**

Six areas were selected according to the geographical regions in China. In each of these areas, we selected 4 distinct groups of subjects (children, adults, pregnant women and lactating women) in regions where the coverage rate of iodised salt was more than 95% and the levels of iodine and fluoride in drinking water were less than or equal to 10 µg/L and 1 mg/L, respectively. We tested the iodine content of salt, urinary iodine (UI), free thyroxin (FT4), thyrotropin (TSH), thyroglobulin (Tg), thyroglobulin antibody (Tg-Ab) and antimicrosomal antibody (TM-Ab) in the 4 groups, and examined the thyroid volume in children.

**Results:**

The median urinary iodine (MUI) concentrations were 271.4 μg/L, 260.2 μg/L, 205.9 μg/L and 193.9 μg/L in children, adults, pregnant women and lactating women, respectively; MUI in children and adults were more than adequate. The goitre prevalence (GP) in children was 6.70%. The odds ratios (OR) of subclinical hypothyroidism in the Tg-Ab- or TM-Ab-positive groups were 3.80, 7.65, 2.01 and 7.47 for children, adults, pregnant women and lactating women, respectively, compared with the negative groups.

**Conclusions:**

The iodine status in children and adults is above the requirement, we should reduce their iodine intake. Subclinical hypothyroidism easily occurs in the Tg-Ab or TM-Ab positive groups.

## Introduction

Iodine deficiency disorders (IDD) are attributed to inadequate iodine intake from foods or drinks, which could cause a low level of thyroid hormones in the body [1,2]. Globally, in 2011, 32 countries and 1.88 billion people remained iodine-deﬁcient, including 241 million schoolchildren, who had insufﬁcient dietary iodine intake [3]. In China, IDD were mainly distributed in 30 provinces (autonomous regions, municipalities) and Xinjiang Production and Construction Corps, with a threatened population of approximately 370 million in the 1970s, including 35 million endemic goitres and 250,000 cases of endemic cretinism [4]. 

Salt iodisation has been recognised as the most effective and cost-efficient strategy to prevent IDD because salt is consumed daily by everybody and by all age groups [5]. Universal Salt Iodisation (USI) was conducted in China from October 1994. Since October 2000, the iodisation level was set at 35 mg/kg.According to the 2005 China National Iodine Deficiency Disorders Surveillance Report, median urinary iodine (MUI) in children aged 8-10 years was 246.3 µg/L; urinary iodine (UI) of children of the same age group stood at 100-200 µg/L in 9 provinces, 200-300 µg/L in 16 provinces, below 100 µg/L in 2 provinces and over 300 µg/L in 5 provinces [6]; these data appeared to have reached national IDD control standards.

Presently, the IDD surveillance is mainly aimed at children aged 8-10 years. However, it is equally important to monitor the iodine status and the thyroid function of adults, pregnant women and lactating women in iodine-replete areas. The relationship between subclinical hypothyroidism and thyroid antibody has been rarely investigated. The uniform iodized salt criterion might not work across China. Each province should adjust iodine level in salt according to their actual situation. Since 2012, China has adopted a new iodized salt standard of 25 or 30 mg/kg (previously the standard was 35 mg/kg), according to the actual situation in each province (autonomous region, municipality) [7]. We aimed to determine whether the new iodised salt criterion produced an impact on these groups of people, with regard to their iodine status and thyroid function. This study was carried out in 2009 to provide baseline data before reducing the iodine concentrations in iodised salt. 

## Material and Methods

**Figure 1:**
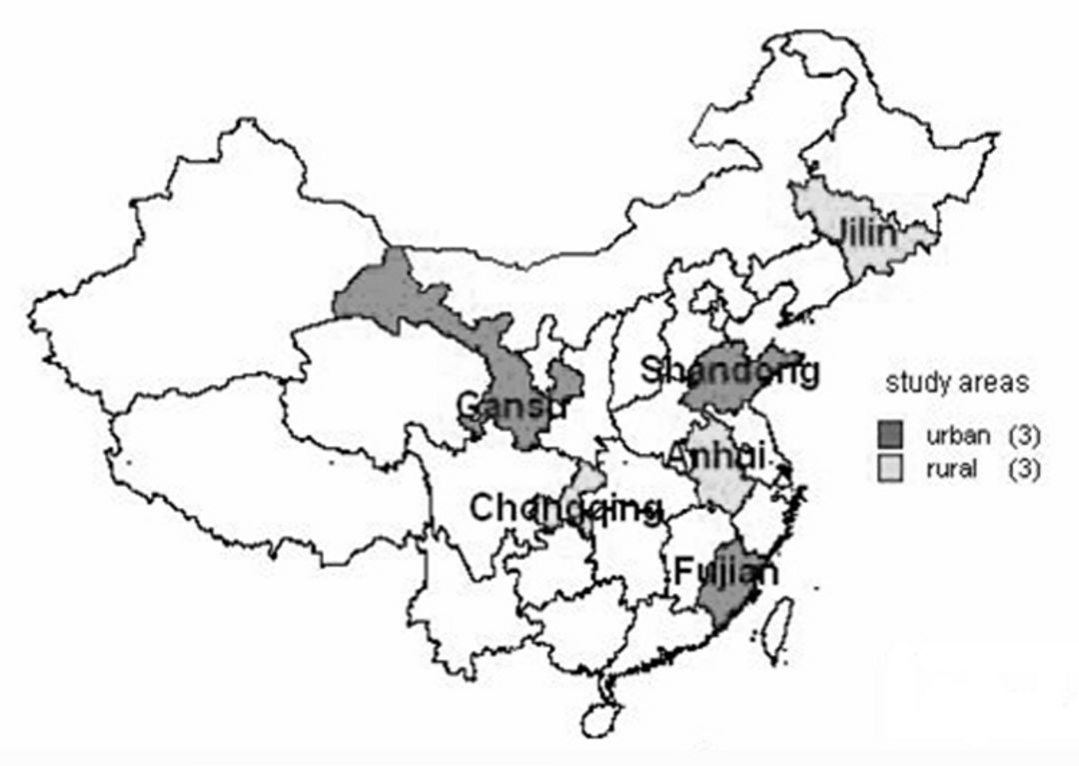
Location of study areas in the map of China. The urban study areas were selected from Gansu, Shandong and Fujian provinces; the rural study areas were selected from Jilin, Anhui and Chongqing provinces. According to geographical regions of China, Fujian and Chongqing are in the southern, Shandong and Anhui are in the middle, Gansu and Jilin are in the northern.

Study areas: China was divided into 6 types of areas according to geographical regions: the southern urban and rural (the south of Yangtse River); the northern urban and rural (the north of Yellow River); the central urban and rural (the areas between the Yangtse River and the Yellow River). We selected 1-2 subdistricts in each urban site and 1-3 townships in each rural site. The study criteria were set as follows: with a middle level of economic development, over 95% of the households should have access to iodised salt; the iodine concentration of drinking water should be below 10 µg/L; the fluoride concentration of drinking water should be less than or equal to 1 mg/L. The selected 6 locations are shown in the map of the China in Figure 1.

In each survey area, 100 school children aged 8-10 years (both sexes), 100 adults aged 18-45 years (both sexes), 50 pregnant women and 50 lactating women were selected either from the local elementary school or community. Sample size selection was based on UI variation [8]. All of the residents who had been residing in the study areas for more than 12 months were considered eligible for inclusion in our study. Those who had thyroid diseases or were taking anti-thyroid drugs at the time of the study were not covered. The survey was carried out from March to December 2009. 

Blood samples (5 to 6ml) were collected from the cubital vein of the forearm and stored in a clean glass tube or plastic pipe; the serum samples were prepared by centrifugation (3000 r/m, 15-20 mins) for half an hour after blood collection, and the aliquots were frozen at -20°C until thyroid hormones could be analysed. All of the blood samples from our subjects in six provinces were tested by the Institute of Endocrinology, Tianjin Medical University. By using toluene as a preservative, casual urine samples were collected from all of the subjects in screw-capped plastic bottles and refrigerated at 4°C; urinary iodine concentrations were tested by provincial-level laboratory in the six provinces/municipality within 1-2 months.

Thyroid volume estimation: Using a portable ultrasound machine with a 7.5-MHz transducer, experienced radiologists from the local Centers for Disease Control and Prevention measured the thyroid volumes of school-aged children (8-10 years) in the six provinces. The thyroid lobe volume was calculated by measuring the depth (d), the width (w) and the length (l) of each lobe by the formula: V (ml) = 0.479 × d × w × 1 (mm)/1000. The sum of both lobes therefore registered the thyroid volume. The volume of the isthmus is excluded. The thyroid glands were classified as enlarged using the reference of the WHO/UNICEF/ICCIDD criteria (2007): the thyroid volumes were defined as goitre of boy over 3.71 ml, 4.19 ml and 4.73 ml and goitre of girl over 3.76 ml, 4.32 ml and 4.98 ml in children aged 8, 9 and 10 years old [1].

Laboratory analysis: The serum free thyroxin (FT4) and thyrotropin (TSH) were measured by chemiluminescent immunoassay (Bayer ADVIA Cetaur System, Bayer Healthcare, Germany); thyroglobulin (Tg), thyroglobulin antibody (Tg-Ab) and antimicrosomal antibody (TM-Ab) were measured by radioimmunoassay (RIA) (Beijing Atom High Tech Co., Ltd); iodine content of salt was determined by the titration method (GB/T 13025.7-1999) [9]; UI was measured by the acid digestion method, a national standard method developed by the China’s Ministry of Health (WS/T107-2006) [10]; and the internal quality control samples of UI were provided by China National Iodine Deficiency Disorders Reference Laboratory. In children and adults, MUI concentrations of between 100 µg/Land 299 µg/L define a population which has no iodine deficiency. In pregnant women and lactating women, MUI concentrations should be controlled between 150 µg/L and 249 µg/L [1]. As far as FT4 was concerned, the normal reference values in whole blood were 10.6-20.9 pmol/L, 11.5-23.5 pmol/L, and 9.2-21.0 pmol/L in children, adult and lactating women, and pregnant women, respectively; and for TSH, the normal reference values were 0.80-5.40 mIU/L, 0.3-5.0 mIU/L, and 0.03-4.54 mIU/L in children, adults and lactating women, as well as pregnant women, respectively. The Tg status, Tg-Ab and TM-Ab reference values were classified as <25 ng/mL, <30% and <25%, respectively, in the 4 groups [11-13].

Statistical analysis: Software such as SPSS (version 10.0) and Excel was adopted for data analysis. While medians were used to describe the UI, breast milk iodine concentrations, FT4, TSH and Tg, mean value was used to describe the iodine content of salt. Chi square test was used to compare prevalence of subclinical hypothyroidism, Tg-Ab and TM-Ab positive rate, prevalence of subclinical hypothyroidism in antibody positive and negative groups. The results were considered statistically significant when P<0.05.

Ethical approval: The study was approved by the ethics committee of the Harbin Medical University and by each local Centre for Disease Control and Prevention. Signed consents in writing were obtained from the participants who agreed to participate in the study. We signed informed consent from the guardians on the behalf of the children participants.

## Results

The effective sample sizes totalled 627, 699, 326 and 332 for children, adults, pregnant women and lactating women, respectively. The basic information of the six areas was nearly satisfied with demand: the level of iodised salt was over 95%; and iodine concentrations of drinking water were below 10 µg/L except Anhui province, and fluoride levels in drinking water were less than or equal to 1 mg/L. The results are shown in Table 1.

**Table 1:** General information of the six survey districts.

Study areas	Coverage of iodized salt (%,)	Iodine in drinking water(µg/L,)	Fluoride in drinking water (mg/L,)
Fujian	100	2.70	0.10
Chongqing	95	1.50	1.00
Shandong	99	2.00	0.10
Anhui	100	10.60	0.53
Gansu	99	0.14	0.22
Jilin	100	2.72	not detected

The six study areas satisfied with demand: the level of iodized salt was over 95%; and iodine concentrations of drinking water were below 10 µg/L except Anhui province, and fluoride levels in drinking water were less than or equal to 1 mg/L.

### Iodine status in 4 groups of subjects

**Figure 2:**
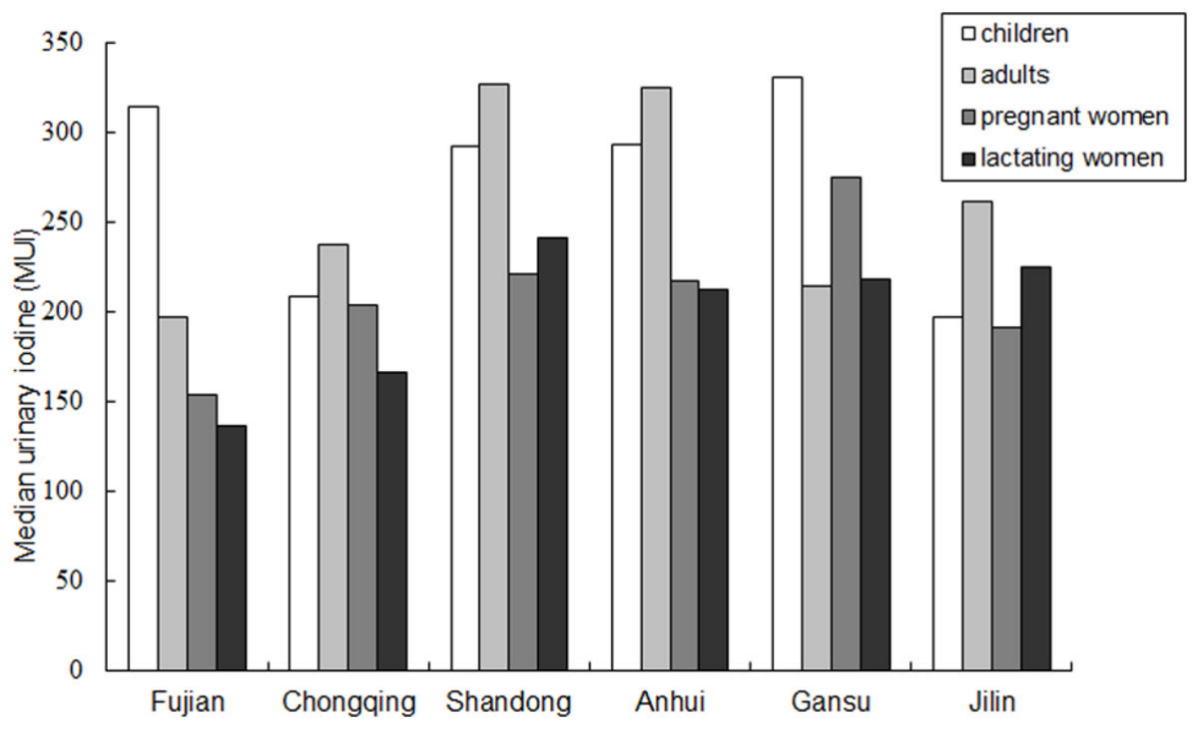
MUI of the six study areas in the 4 groups of population. Of the 6 provinces, MUI in children was above the requirement except Jilin province; MUI in adults was above the requirement except Fujian province; And, MUI in lactating women was above the requirement except Fujian and Chongqing provinces. MUI was adequate in pregnant women of the six provinces.

The iodine status of school-aged children, adults, pregnant women and lactating women is provided in Table 2. The results revealed that the MUI values of children and adults were above the requirement levels (100-200 µg/L), only 2.89% of children and 5.67% of adults had a urinary iodine concentration (UIC) below 100 µg/L, and approximately 40.03% of children and 35.41% of adults had a UIC value standing at over 300 µg/L. Pregnant women and lactating women had values within the range of adequacy. The MUI values of the six provinces are depicted in Figure 2. Of the 6 provinces, MUI in children was above the requirement except Jilin province; MUI in adults was above the requirement except Fujian province; And, MUI in lactating women was above the requirement except Fujian and Chongqing provinces. MUI was adequate in pregnant women of the six provinces. The iodine content of table salt for all of the 4 groups was found within the recommended standard (20-50 mg/kg). The total goitre prevalence (GP) was 6.70% (42 of 627) in children. The median values of FT4, TSH and Tg of the 4 groups were all within the reference range. The total Tg-Ab positive rate was 2.55% (16 of 627), 12.44% (87 of 699), 8.28% (27 of 326) and 7.53% (25 of 332) and the total TM-Ab positive rate was 2.87% (18 of 627), 9.16% (64 of 699), 6.13% (20 of 326) and 5.42% (18 of 332) in children, adults, pregnant women and lactating women, respectively, no statistical difference was found in the high MUI provinces and low MUI provinces. The Tg-Ab and TM-Ab positive rates in the adults ran up to 12.44% and 9.16% respectively, which were highest in the 4 groups.

**Table 2:** Iodine status in the 4 groups of population.

Group	No.	Age	Male/ female	MUI (µg/L)	Iodine concentrations in iodized salt (mg/kg)	Iodine in breast milk (µg/L)	GP (%)	FT4 (pmol/L)	TSH (mIU/L)	Tg (ng/ml)	Tg-Ab positive rate (%)	TM-Ab positive rate (%)
Children	627	9.6±2.3	314/313	271.4	28.6±5.7	—	6.70	16.71	2.79	5.75	2.55	2.87
Adults	699	34.8±7.2	349/350	260.2	28.2±7.1	—	—	16.16	1.77	7.04	12.44	9.16
Pregnant women	326	26.1±4.8	0/326	205.9	31.3±6.7	—	—	13.29	1.74	4.85	8.28	6.13
Lactating women	332	26.3±4.2	0/332	193.9	29.5±6.4	171.2	—	15.04	1.90	5.04	7.53	5.42

Medians were used to describe the iodine in breast milk, FT4, TSH and Tg. MUI in children and adults is above the requirement; the median values of FT4, TSH and Tg of the 4 groups were all within the reference range; significant difference were found in Tg-Ab and TM-Ab positive rate of the 4 groups of population.

### FT4, TSH and Tg abnormal rates in 4 groups of population

The percentage of lactating women was the highest (10.54%) in relation to their FT4 below the reference value. The rate of TSH which stood above the reference value was found in 10.69% of the children, and 3.43% of the adults were discovered with their rate of Tg above the reference value. The results are shown in Table 3. 

**Table 3:** FT4, TSH and Tg abnormal rates in the 4 groups of population.

Group	No.	Low FT4 (%)	High TSH (%)	High Tg(%)
Children	627	1 (0.16)	67 (10.69)	3 (0.48)
Adults	699	8 (1.14)	38 (5.44)	24 (3.43)
Pregnant women	326	1 (0.30)	13 (3.99)	5 (1.53)
Lactating women	332	35 (10.54)	17 (5.12)	4 (1.20)

Low, below the reference value; High, above the reference value.

%, frequency.

### Relationship between subclinical hypothyroidism rate and the antibody

Subclinical hypothyroidism was defined as TSH level above reference range and the FT4 level within the normal reference value. It was detected that the subclinical hypothyroidism rate in the Tg-Ab or TM-Ab positive group was higher than in the negative group. A significant difference was found in children, adults and lactating women (χ^2^=32.78, p=0.01; χ^2^=32.78, p<0.001; χ^2^=12.17, p<0.001). Compared with the Tg-Ab or TM-Ab negative groups, the odds ratios (OR) of subclinical hypothyroidism in the Tg-Ab or TM-Ab positive groups were 3.80 (1.35, 10.68), 7.65 (3.81, 15.34), 2.01 (0.40, 10.13) and 7.47 (2.41, 23.13) for children, adults, pregnant women and lactating women, respectively. The subclinical hypothyroidism rate in the high MUI provinces compared to low MUI provinces, no statistical difference was found in children, adults, pregnant women and lactating women. The total subclinical hypothyroidism rate appeared to be at 10.53% (66 of 627), 4.72% (33 of 699), 3.99% (13 of 326) and 4.22% (14 of 332) in children, adults, pregnant women and lactating women respectively (Table 4).

**Table 4:** Subclinical hypothyroidism rate in the antibody positive and negative group of 4 groups of population.

	Subclinical hypothyroidism rate (%)	
Group	Tg-Ab or TM-Ab positive	Tg-Ab and TM-Ab negative
Children	7/24 (29.17) **^*▽*^**	59/603 (9.78)
Adults	16/89 (17.98) **^*▽*^**	17/610 (2.79)
Pregnant women	2/28 (7.14)	11/298 (3.69)
Lactating women	5/27 (18.52) **^*▽*^**	9/305 (2.95)

^▽^ Compared with antibody negative groups (p<0.05).

### TSH levels in different urinary iodine

**Figure 3:**
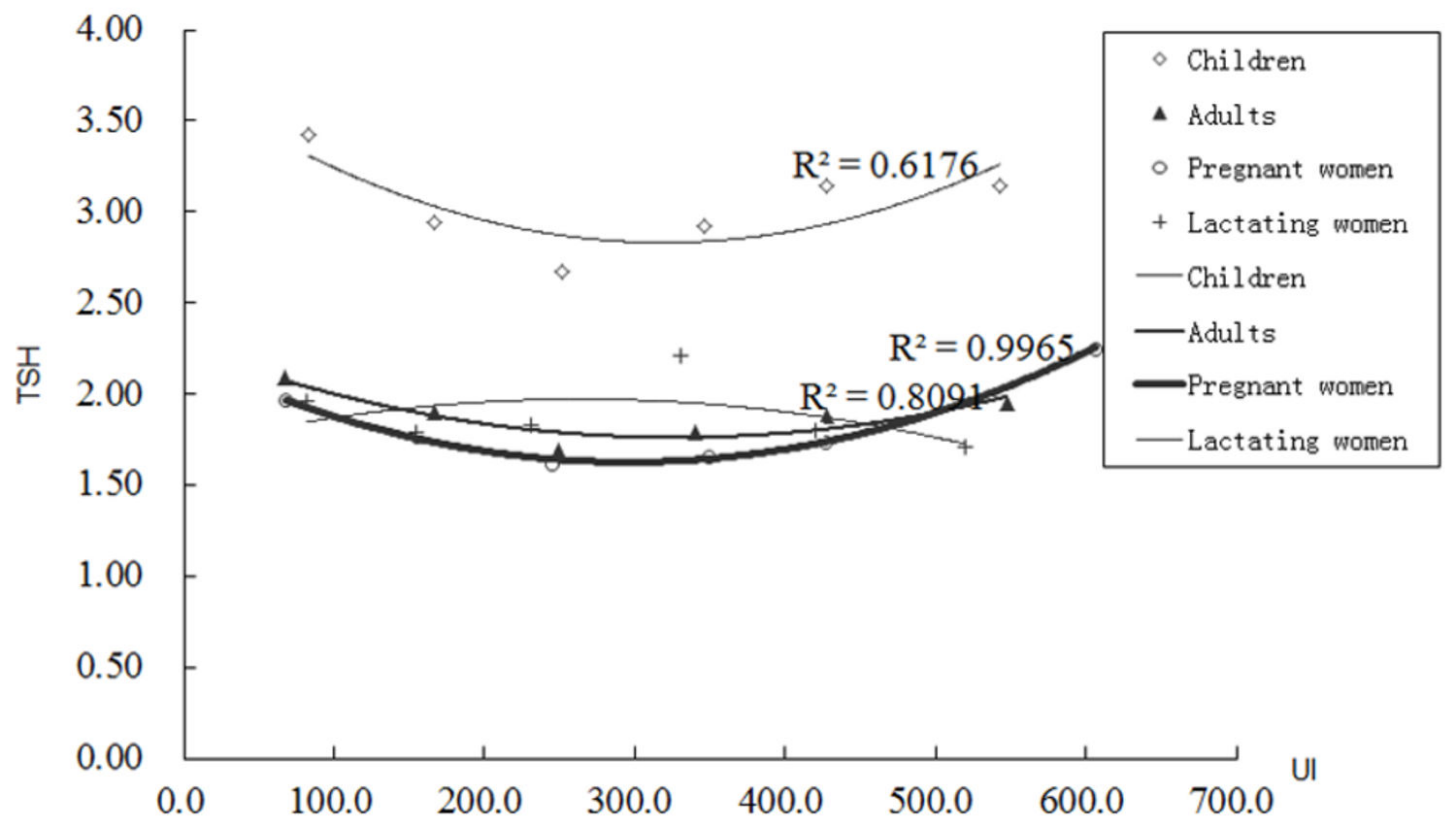
MUI and TSH relationship in 4 groups of population. The MUI was divided into 6 groups (<100, 100-199.9, 200-299.9, 300-399.9, 400-499.9 and >500 µg/L). The TSH value was spotted to decrease and subsequently increase with an elevation in MUI, and formed a “U curve” relationship in children, adults and pregnant women except in lactating women. The R^2^ was 0.6176, 0.8091 and 0.9965 in children, adults and pregnant women respectively.

The UI values were divided into 6 groups (<100, 100-199.9, 200-299.9, 300-399.9, 400-499.9 and >500 µg/L); the MUI and TSH values were calculated in each group (Table 5 and Figure 3). The TSH value was spotted to decrease and subsequently increase with an elevation in UI, except in lactating women. In 200-299.9 µg/L groups, the TSH value was lowest in children, adults and pregnant women.

**Table 5:** UI and TSH relationship in different UI levels in 4 groups of population.

	Children			Adults			Pregnant women			Lactating women	
UI levels	MUI (µg/L)	TSH (mIU /L)		MUI (µg/L)	TSH (mIU /L)		MUI (µg/L)	TSH (mIU /L)		MUI (µg/L)	TSH (mIU /L)
<100	83.4	3.42		67.5	2.09		67.9	1.97		81.9	1.96
100-199.9	166.6	2.94		167.4	1.90		154.5	1.77		154.1	1.79
200-299.9	251.8	2.67		249.1	1.69		244.4	1.62		231.7	1.83
300-399.9	346.8	2.93		340.2	1.79		348.3	1.66		330.9	2.22
400-499.9	428.1	3.14		427.6	1.88		426.7	1.74		420.1	1.80
>500	542.7	3.15		547.4	1.95		606.2	2.26		520.4	1.71

The median values of UI and TSH were calculated in each group. The TSH value decreased and subsequently increased with the elevation of UI in children, adults and pregnant women.

## Discussion

Our organization is responsible for the national IDD surveillance work. Presently, we have not found any IDD surveillance report which combines FT4, TSH and Tg testing in conjunction with UI; we therefore aim to assess the iodine nutrition of the 4 groups of population in a comprehensive fashion. A wide variety of target groups, including newborns, infants, preschool children, school-aged children, and certain groups of adults might serve as the focus for IDD surveillance. However, the representativeness and accessibility of some groups vary, so we selected the 4 groups to represent the general population. The 6 sampled areas had similar living conditions (economic level,coverage of iodised salt, iodine and fluoride concentrations in drinking water), we combined the subjects to evaluate the present iodine nutrition condition in China. According to the WHO’s recommendation, the following indicators were adopted to assess iodine status, monitor and evaluate the impact of salt iodisation on the population: MUI, GP, serum TSH and Tg [1]. However, given the limitations of these indicators, we added FT4, Tg-Ab and TM-Ab as new indicators. 

From the time that USI was launched in October 1994, we have been observing the progress in IDD control and prevention in China. Presently, children and adults exhibit more than adequate levels of iodine nutrition, the children’s GP has risen to 6.70% (42 of 627). According to the WHO/UNICEF/ICCIDD criteria, it poses a threat the public health when the GP exceeds 5% [1]. Fluoride, a goitrogenic substance in drinking water, is another contributing factor to high GP. The fluoride concentration of drinking water was as high as 1.00 mg/kg in Chongqing municipality, which led Chongqing to have the highest GP (18.37%, 18 of 98) amongst all study areas. UIC was found to be above the requirement or at excessive level, which was likely responsible for the possible risk of thyroiditis, hyperthyroidism, hypothyroidism, and goiter [14]. 

It was unveiled that the FT4 and UI levels were low in lactating women and pregnant women. Taking into account iodine loss through breast milk (171.2 µg/L) in lactating women, the UI remained lower than in the other 3 groups of population. The total rate which stood below the FT4 reference value was detected in 10.54% of the lactating women, which was higher than in the other 3 groups. The iodine deficiency results reduced reproductive success in women and produced a low FT4 level and hypothyroxinemia in several cases. At the time of the study, the IDD surveillance in China did not include the FT4 level screening indicator in pregnant women and lactating women. However, it was believed that FT4 screening might be a more sensitive index for monitoring IDD, assess iodine nutrition and prevent hypothyroxinemia. The TSH value decreased and subsequently increased with the elevation of UI, except in lactating women (part of iodine was lost through breast milk excretion), and formed a “U curve” relationship in children, adults and pregnant women. Subclinical hypothyroidism was likely to occur more easily in the populations with iodine intakes above and below the reference range. The median of TSH level was 2.79 mIU/L in children, which was higher than the other 3 groups of population. The rate of subclinical hypothyroidism was also higher in children than in the other 3 groups of population. When UI stood at 200-299.9 µg/L, the TSH value was found lowest in children, adults and pregnant women. So should subclinical hypothyroidism prevention is our goal, the UI level probably needs to be controlled under 300 µg/L. Some research found that TSH level from 1.0 to 1.9 mIU/L was associated with the lowest subsequent incidence of abnormal thyroid function [15]. Several research findings have documented that subclinical hypothyroidism might be associated with cardiovascular risk factors and a more atherogenic lipid profile in children [16-18]. In the six survey areas, children were discovered with more than adequate UI levels. While salt iodisation played a key role in the control and prevention of IDD, additional research is warranted to evaluate whether salt iodisation might contribute to the increase of patients with subclinical hypothyroidism.

Tg, in comparison with TSH, is a more sensitive indicator of iodine repletion [19,20], and therefore is a key target to evaluate thyroid function [21-24]. The Tg level in adults and the rate of above the reference value were higher than in the other 3 groups. Adults were also found with higher Tg-Ab and TM-Ab positive rates than the other 3 groups. Compared with the antibody negative group, the subclinical hypothyroidism detection rate was higher in positive group of the 4 groups of population. Especially in adults, the OR was as high as 7.65. The serum Tg was a possible factor that increased the goitre prevalence, thereby resulting in thyroid inflammation or damage. The high Tg level may increase the numbers of patients with thyroid or other autoimmune disorders. At the time of the study, the UI of adults was more than adequate; a debate was underway with regard to how the excessive consumption of iodised salt might cause an increase in thyroid disease. Great attention has been paid to people’s health consciousness and improvement in health detection technology. 

We were proud that the FT4, TSH, Tg, Tg-Ab and TM-Ab testing was combined with UI to monitor IDD, iodine status and thyroid function in the 4 groups of population. However, several limitations resided in this study. For example, the thyroid size was not measured by one person in the six areas. The UI was estimated in a spot urine sample and not in a 24-hour collected sample, the iodine levels in drinking water and urine were measured by each local Centre for Disease Control and Prevention, and the iodine concentrations in drinking water of Anhui province was slightly higher than 10 µg/L. Subjects were not systematically recruited, posing some selection bias to the study. The pregnant women were not divided into 3 trimesters. Anti-TPO antibody, which is a better marker than TM-Ab of autoimmunity, was not assessed.

In this cross-sectional survey, UI levels were found to be above the normal requirements in children and adults; the iodine nutrition of pregnant women and lactating women had reached an adequate level. We should therefore reduce the iodine intake by children and adults and pay greater attention to a high individual risk of iodine-induced hyperthyroidism and subclinical hypothyroidism caused by excessive iodine intake in genetically susceptible individuals of autoimmune thyroid diseases. The Tg-Ab and TM-Ab positive rates were a risk factor that caused subclinical hypothyroidism in children, adults and lactating women. Since 2012, China has adopted a new iodized salt standard of 25 or 30 mg/kg selected by provinces (previously the standard was 35 mg/kg). This study will provide important baseline information to monitor the iodine nutrition and thyroid function before and after the implementation of this new standard. 
